# Supplementary material for: Telomerase and Pluripotency Factors Jointly Regulate Stemness in Pancreatic Cancer Stem Cells
Source: Cancers (Basel). 2021 Jun 23;13(13):3145. doi: 10.3390/cancers13133145 (PMC8268125; doi:10.3390/cancers13133145)
Supplement: Supplementary file 1 [file cancers-13-03145-s001.zip › cancers-1228733-SI.pdf]

Telomerase and Pluripotency Factors Jointly Regulate Stemness in Pancreatic Cancer Stem Cells

Karolin Walter [1], Eva Rodriguez-Aznar [1], Monica S. Ventura Ferreira [2], Pierre-Olivier Frappart [1,3], Tabea Dittrich [1], Kanishka Tiwary [1], Sabine Meessen [4], Laura Lerma [5], Nora Daiss [1], Lucas-Alexander Schulte [1], Zeynab Najafova [6], Frank Arnold [1], Valentyn Usachov [1], Ninel Azoitei [1], Mert Erkan [7,8], Andre Lechel [1], Tim H. Brümmendorf [2], Thomas Seufferlein [1], Alexander Kleger [1], Enrique Tabarés [5], Cagatay Günes [4], Steven A. Johnsen [9], Fabian Beier [2], Bruno Sainz, Jr [10,11,12] and Patrick C. Hermann [1,*]

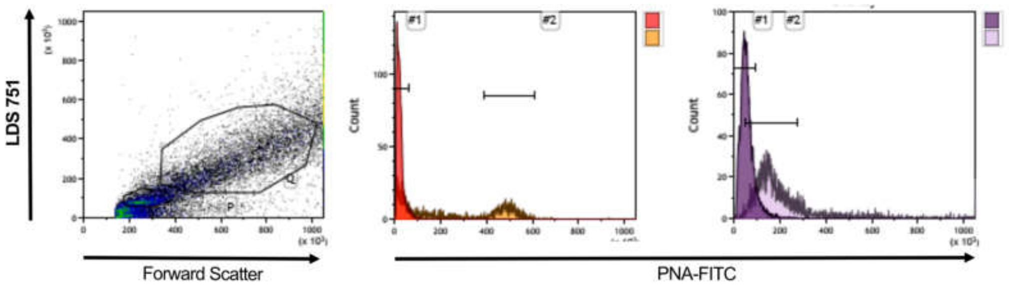

Figure S1. Representative flow-Fish of PDAC cell lines.

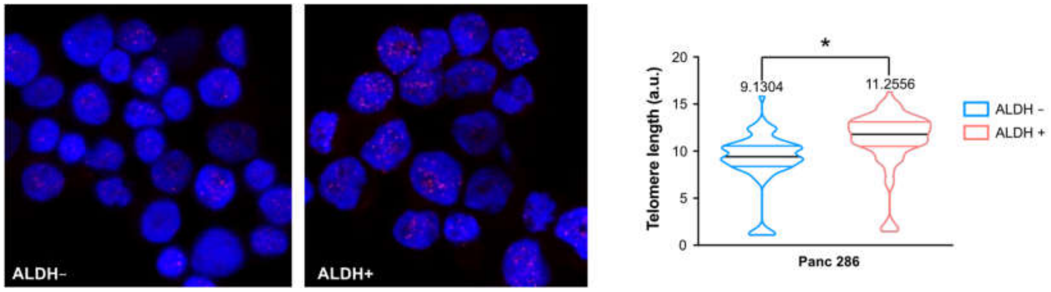

Figure S2. Telomerase activity stabilizes telomere length in pancreatic CSCs. Representative pictures and Q-FISH telomere length analysis in Aldefluor positive and negative cells (40x magnification is shown). The mean is depicted in numbers and as black line, >150 measurements per group. Data are represented as mean ± SEM. *$p \leq 0.05$ (Mann-Whitney-U test).

Figure S3

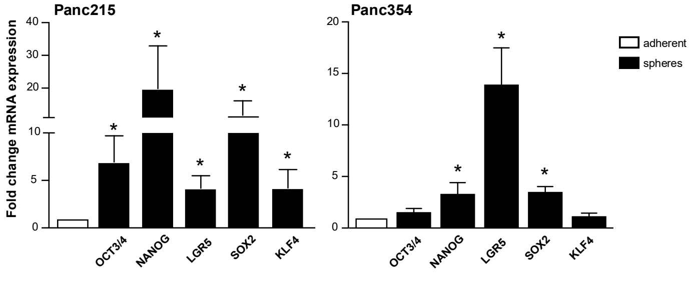

Figure S3. RT-qPCR analysis of pluripotency/stemness-associated genes in spheres and adherent cell culture conditions. Data are represented as mean ± SEM, $n = 4$ independent experiments, *$p \leq 0.05$ (Mann-Whitney-U test).

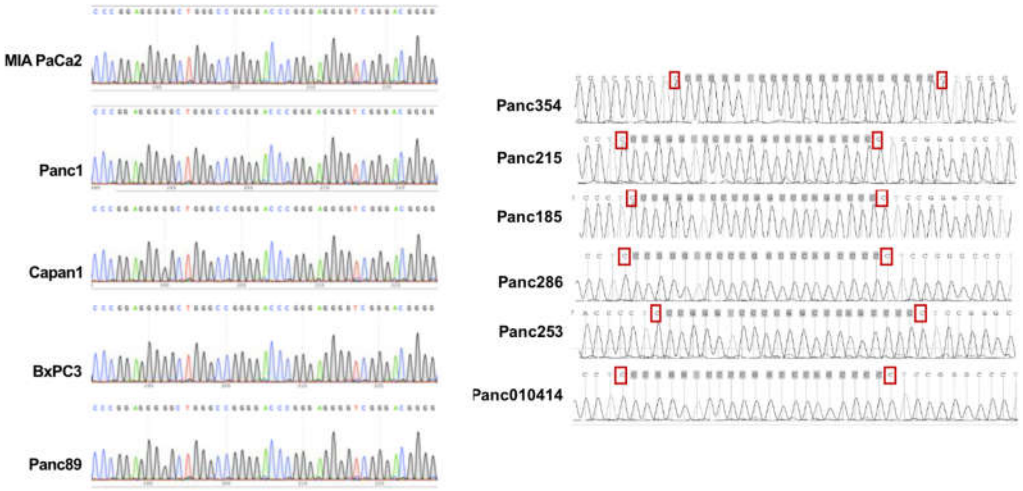

Figure S4. TERT promoter mutation analysis of C250T and C228T sites in five established pancreatic cancer cell lines (MIAPaCa2, Panc1, Capan1, BxPC3 and Panc89) and six primary pancreatic cancer cell lines (Panc354, Panc215, Panc185, Panc286, Panc253 and Panc010414).

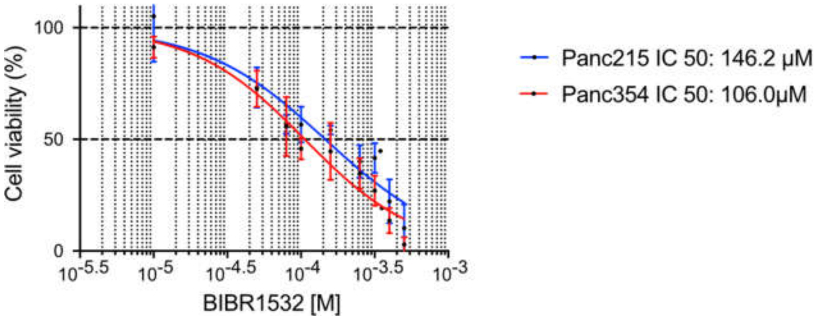

Figure S5. Targeting telomerase activity with BIBR1532. MTT assay to determine the respective IC50 of BIBR1532 in the utilized primary pancreatic cancer cells.

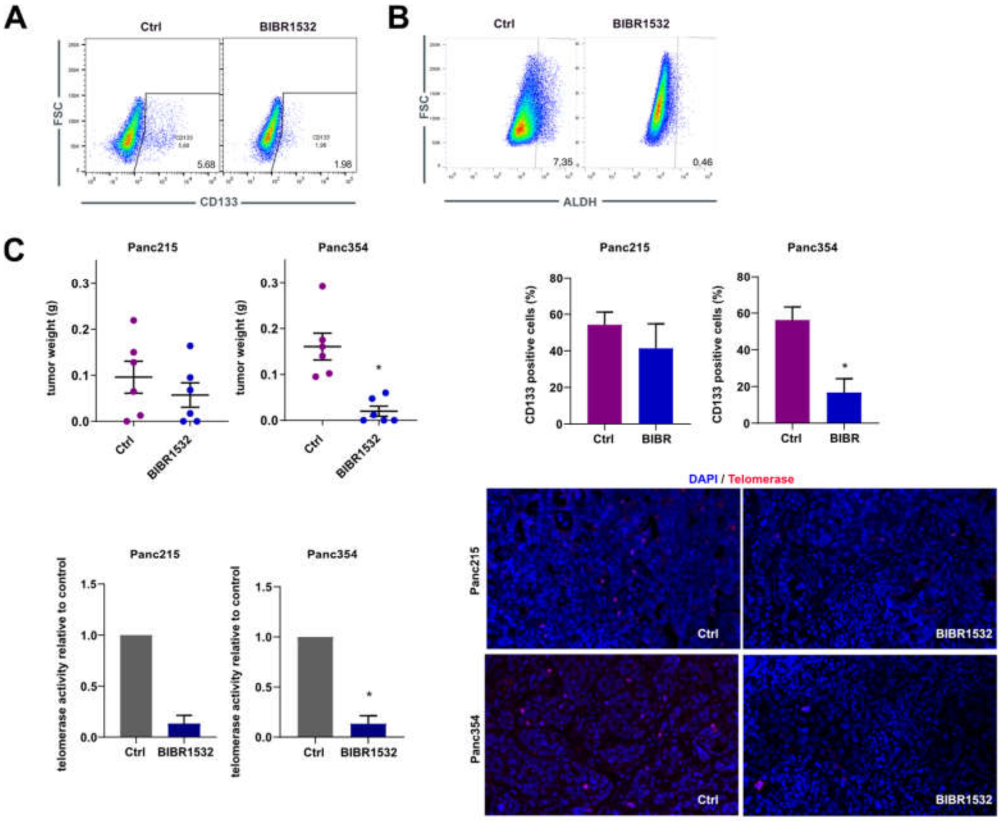

Figure S6. Telomerase inhibition as treatment strategy for pancreatic cancer (stem) cells. Representative cytometry illustrations of (**A**) CD133 and (**B**) Aldefluor positive cells after vehicle (Ctrl) or BIBR1532 treatment. (**C**) Tumor weight, CD133 flow cytometry analyses, telomerase activity and telomeres staining of Ctrl and BIBR1532 pre-treated tumors, 10x magnification is shown.

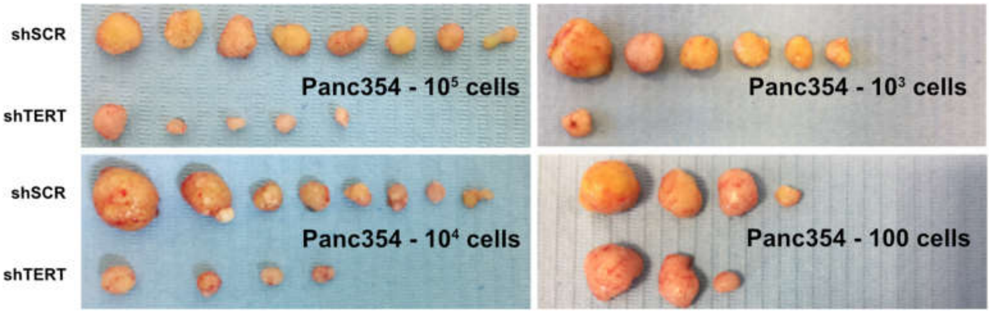

Figure S7. Knockdown of TERT diminishes pancreatic cancer growth. Representative pictures of explanted subcutaneous tumors from Panc354 ELDA assays.

Image Figure 1G

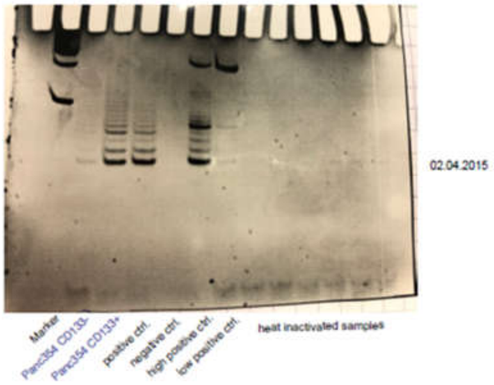

Image Figure 1I

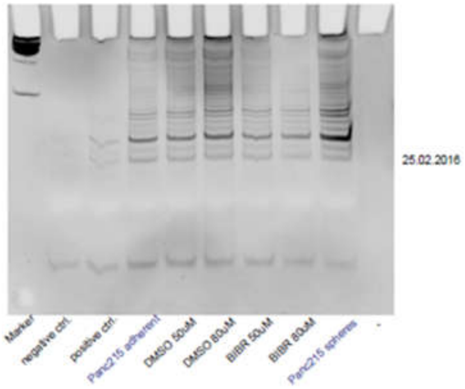

Detailed information about Figure 1.

Table S1. Antibodies.

Antibody	Application	Manufacturer
α-hu-CD133/1-(AC133)-APC	FC	MiltenyiBiotec (Cat no. 130090826)
α-hu-CD133/1-(AC133)-PE	FC	MiltenyiBiotec (Cat no. 130080801)
α-hu-CD133/1-(AC133)-PE-VIO770	FC	MiltenyiBiotec (Cat no. 130102358)
α-hu-EpCAM-APC	FC	BD Biosciences (Cat no. 347200)
α-γ-H2A.X (Ser139)	IF	Merck (Cat no. 05-636)

Table S2. RT qPCR Primers.

Primer	Sense	Antisense	Manufacturer
HPRT			Qiagen (Cat no. QT000590669)
NANOG			Qiagen (Cat no. QT01025850)
LGR5			Qiagen (Cat no. QT00027720)
TERT			Qiagen (Cat no. QT000734099)
TRF1			Qiagen (Cat no. QT00059528)
OCT3/4	TGAACCTCAGC-TACAAACAGGTG	AACTGCATGCAGGACTG-CAGAG	ThermoFisher
KLF4	ACCCACACAGGTGAGAAACC	ATGTGTAAGGCGAGGTGGTC	ThermoFisher
SOX2	AGAACCCCAAGATGCACAAC	CGGGCCGGTATTTATAATC	ThermoFisher
ACX	GCGCGGCTTA(CCCTTA)$_3$CCCTAA		SigmaAldrich
TS	AATCCGTCGAGCAGAGTT		SigmaAldrich
